# Safety Considerations in the Development of Hippo Pathway Inhibitors in Cancers

**DOI:** 10.3389/fcell.2019.00156

**Published:** 2019-08-14

**Authors:** Satoko Kakiuchi-Kiyota, Melissa M. Schutten, Yu Zhong, James J. Crawford, Anwesha Dey

**Affiliations:** ^1^Department of Safety Assessment, Genentech, Inc., South San Francisco, CA, United States; ^2^Department of Discovery Chemistry, Genentech, Inc., South San Francisco, CA, United States; ^3^Department of Discovery Oncology, Genentech, Inc., South San Francisco, CA, United States

**Keywords:** Hippo pathway, drug development, target safety assessment, genetically engineered mouse models, regenerative medicine

## Abstract

The Hippo pathway is a critical regulator of cell and organ growth and has emerged as a target for therapeutic intervention in cancers. Its signaling is thought to play an important role in various physiological processes including homeostasis and tissue regeneration. To date there has been limited information about potential pharmacology-related (on-target) safety liabilities of Hippo pathway inhibitors in the context of cancer indications. Herein, we review data from human genetic disorders and genetically engineered rodent models to gain insight into safety liabilities that may emerge from the inhibition of Hippo pathway. Germline systemic deletion of murine Hippo pathway effectors (Yap, Taz, and Teads) resulted in embryonic lethality or developmental phenotypes. Mouse models with tissue-specific deletion (or mutant overexpression) of the key effectors in Hippo pathways have indicated that, at least in some tissues, Hippo signaling may be dispensable for physiological homeostasis; and appears to be critical for regeneration upon tissue damage, indicating that patients with underlying comorbidities and/or insults caused by therapeutic agents and/or comedications may have a higher risk. Caution should be taken in interpreting phenotypes from tissue-specific transgenic animal models since some tissue-specific promoters are turned on during development. In addition, therapeutic agents may result in systemic effects not well-predicted by animal models with tissue-specific gene deletion. Therefore, the development of models that allows for systemic deletion of Yap and/or Taz in adult animals will be key in evaluating the potential safety liabilities of Hippo pathway modulation. In this review, we focus on potential challenges and strategies for targeting the Hippo pathway in cancers.

## Introduction

**FIGURE 1:**
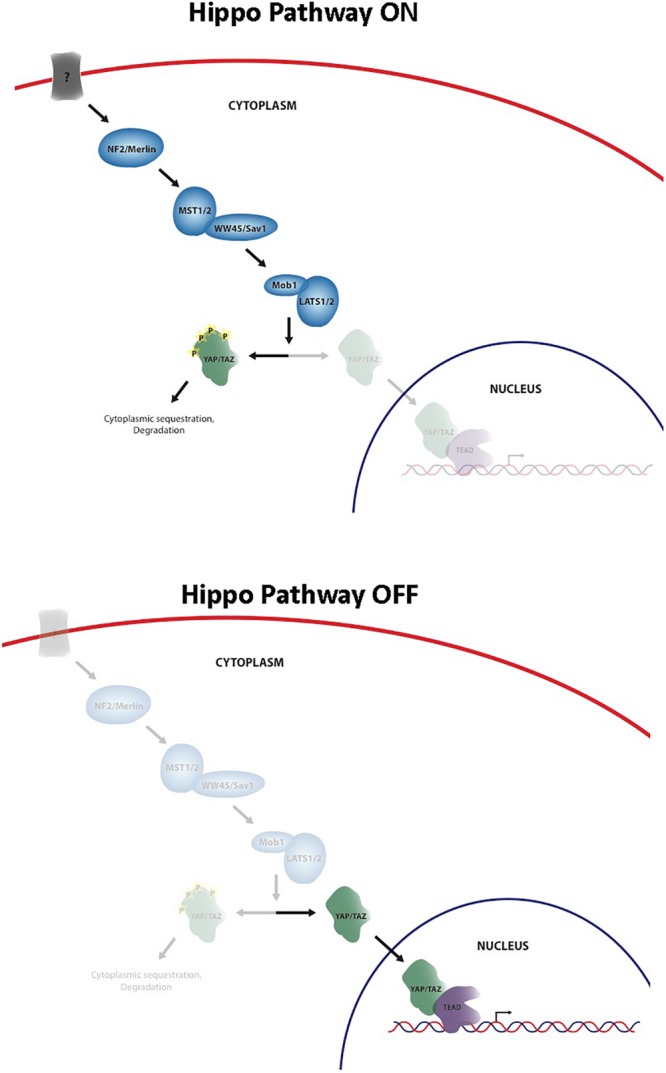
Schematic of the core Hippo pathway.

The Hippo signaling pathway has been identified as a key regulator of cell and organ growth and various physiological processes including normal homeostasis and tissue regeneration. The pathway was originally discovered in *Drosophila melanogaster* and since that time an increasing body of knowledge has been generated about the importance of this pathway in both normal development and disease states, such as cancer. Core elements of the pathway are highly conserved from *Drosophila* to mammals, including Merlin homolog, neurofibromin 2 (NF2), two Hpo homologs (Mst1 and Mst2), one Sav homolog (WW45 or Sav1), two Wts homologs (Lats1 and Lats2), and two Mats homologs (MOBKL1A and MOBKL1B, often collectively referred to as Mob1), Yki homologs, Yes-associated protein (YAP) and transcriptional co-activator with PDZ-binding motif (TAZ, also known as WWTR1), and Scalloped homolog, transcriptional enhancer associate (TEA) domain family members (TEADs) (Pan, 2010) (Figure 1). Unlike *Drosophila* that contains one TEAD gene, mammals have four TEAD genes (TEADs 1–4), all of which have conserved TEA DNA binding domain and YAP binding domain (Holden and Cunningham, 2018). YAP and TAZ share approximately 50% amino acid sequence identity with a similar domain organization, and each contains a TEAD binding domain (Moroishi et al., 2015). They are transcriptional coactivators of TEADs, and nuclear YAP/TAZ-TEAD complexes activate expression of target genes that are involved in cell proliferation, apoptosis, differentiation/regeneration, and tissue homeostasis. Subcellular localization of YAP and TAZ is tightly regulated through a phosphorylation-dependent inhibition mechanism; when the pathway is activated, MST1/2 phosphorylate and activate LATS1/2, which in turn directly phosphorylate YAP/TAZ on multiple serine residues, resulting in cytoplasmic retention and sequestration via a 14-3-3 interaction, followed by ubiquitination and degradation (Pan, 2010). In contrast, when YAP/TAZ are not phosphorylated, they are able to translocate into the nucleus by as-yet-unknown means, bind TEAD, and activate transcription of Hippo target genes (Pan, 2010). Regulation of the Hippo pathway requires the integration of a wide variety of positive and negative inputs from the extracellular and intracellular environment.

Recently, the Hippo pathway has been shown to have a role in the development of several different types of cancer, including liver, breast, skin, and colon cancers (Verfaillie et al., 2015; Liu et al., 2016, 2017; Hagenbeek et al., 2018). Overexpression, amplification, and nuclear localization of YAP and TAZ have been demonstrated in many of these human cancers, however, the underlying mechanism of Hippo pathway deregulation, as well as YAP and TAZ activation therein, is not well understood. Given the role of the Hippo signaling pathway in cancer development across these diverse cancer types, the Hippo pathway is an attractive therapeutic target for the treatment of these diseases. When one considers inhibiting the Hippo pathway as a therapeutic approach, it is immediately obvious that inhibiting a number of the pathway members including the core kinase cascade might be problematic. Nf2, Mst1/2, and Lats1/2 are known tumor suppressors, and viable approaches to activate these targets are not readily apparent (Crawford et al., 2018). The most plausible strategy to inhibit the Hippo pathway is by blocking the YAP/TAZ-TEAD interface (Crawford et al., 2018; Calses et al., 2019).

To enable the design of a therapeutic agent to target this pathway, a solid understanding of the regulation of various components of the Hippo pathway will be needed. However, there are gaps in our current knowledge that are essential to fill in order for us to fully understand the clinical implications of targeting this pathway both in terms of clinical efficacy and safety. Current gaps include: (1) strategies to target extracellular cues that modulate pathway activation (on/off transcriptional signals); (2) YAP/TAZ -TEAD association/dissociation with DNA trafficking, and trafficking from the cytoplasm to the nucleus and the subsequent downstream effects of signaling in various tissue types; and (3) the role of Hippo signaling in normal physiology of various organs. In this review, we provide a target safety assessment of the Hippo pathway modulation in the context of cancer indications. Target safety assessments provide insight into the potential safety liabilities that may result from modulating a certain target. This information is used to educate toxicologists and project teams such that target-related liabilities can be identified and addressed early, and specific mitigation strategies can be designed based on the target patient population. We will review the data from human genetic disorders and genetically engineered mouse models to understand potential safety concerns associated with Hippo pathway inhibition.

## Value of Human Genetic Disorders and Animal Models for Safety Considerations During Drug Discovery

Data from spontaneously occurring human genetic variants and genetically engineered rodent models, including gene knockout and knockin models, have been critical tools in safety de-risking of drug development. For some targets, a strong correlation has been shown between rodent phenotypes and human pharmacology, which emphasizes the applicability of these phenotypes to humans (Dambach et al., 2016). Additionally, these animal models can be incorporated into investigation of mechanisms of toxicity. In the early discovery stage, tool compounds are often used to understand pharmacology-mediated, on-target toxicity as well as chemical scaffold-related liabilities. However, the quality of tool compounds in the literature is often questionable, which can lead to conclusions being drawn that are ultimately misleading and unrelated to the target (Workman and Collins, 2010; Blagg and Workman, 2017). Thus, it is important to integrate the outcomes from the dosing of tool compounds and from human genetic disorders and/or animal models and assess the safety of the intended pharmacological target.

For signaling pathways important in development, gene mutations in conventional, germline knockouts often result in embryonic lethality (Dambach et al., 2016). Indeed, mice with germline deletion of murine Yap, Tead1, and Tead4 were embryonic lethal (Schlegelmilch et al., 2011; Bai et al., 2012; Zanconato et al., 2015). Mice with germline deletion of murine Taz were viable but with a low survival rate and abnormal phenotypes associated with kidney and lung development (Hossain et al., 2007; Makita et al., 2008). These data are consistent with the understanding that Hippo signaling is essential for organ growth and development, and these developmental phenotypes do not necessarily imply that targeting the Hippo pathway in cancers is intractable.

To assess the potential safety liabilities of targeting the Hippo pathway in adulthood, it has been beneficial to utilize conditional knockouts where the loxP-flanked gene of interest is deleted by using Cre recombinase (Cre) under the control of a promoter with the desired spatial and temporal pattern of expression (Albanese et al., 2002). Temporally controlled systemic deletion using a tamoxifen– or tetracycline-driven Cre is most desirable since it most-closely mimics the effects of therapeutic agents, bypasses the influence of developmental phenotypes, and deletes a target gene across tissues at a chosen time in adulthood. A doxycycline-inducible shRNA-mediated reversible gene silencing (knockdown) model (Premsrirut et al., 2011) can be also useful. This model enables evaluation of the reversibility of observed phenotypes, which is not possible with genetic deletion of the target (i.e., Cre/loxP system-mediated knockout).

In this review, we summarize data from human genetic disorders as well as various mouse models for the key Hippo pathway effectors (Yap, Taz and Teads) and implications in targeting this pathway in diseases.

## Phenotypic Data Analyses

### Genetic Mutations in Humans

There are some reports of YAP1 or TEAD1-related early defects in embryonic eye development in human. A heterozygous loss-of-function mutation in YAP1 has been reported in individuals from a family with isolated ocular coloboma (Williamson et al., 2014), microphthalmia and/or coloboma (Holt et al., 2017; Oatts et al., 2017). Consistent with ocular findings in humans with YAP1 mutations, Yap1 mRNA/protein expression was detected during the eye development in mouse at embryonic day 9.5 (E9.5) through E12.5 (Williamson et al., 2014). Also, a missense mutation of TEAD1 was identified as the cause of helicoid peripapillary chorioretinal degeneration (Fossdal et al., 2004).

A different nonsense mutation in YAP1 was reported in another family with coloboma as well as non-ocular abnormalities, including hearing loss, intellectual disability, hematuria, and orofacial clefting (Williamson et al., 2014). Yap1 expression and/or function was also reported in mouse embryo during central nervous system (CNS) development, including brain and ear (Tremblay and Camargo, 2012; Varelas, 2014), and craniofacial development (Wang et al., 2016). These results support that non-ocular abnormalities observed in this family were also associated with YAP1 (Williamson et al., 2014). These are likely developmental phenotypes, and the probability of occurrence should be low in patients (Table 1).

**TABLE 1:** Potential target organs of Hippo pathway inhibition by blocking TEAD and YAP/TAZ.

**Organs**	**Rationale**
Liver	• Appears dispensable for physiological homeostasis • No overt hepatic findings in mice overexpressing a dominant negative form of Tead2 (Liu-Chittenden et al., 2012) or polyC injected-Mx1-Cre; Yap^flox/flox^ mice (Bai et al., 2012) • Important for hepatocyte and bile duct regeneration upon tissue damage • Compromised hepatocyte and bile ductular reaction in polyC injected-Mx1-Cre; Yap^flox/flox^ mice after bile duct ligation (Bai et al., 2012) • Inefficient regeneration in Albumin-Cre; Yap^flox/flox^; Taz^flox/flox^ mice following two-third partial hepatectomy (Lu et al., 2018)
Gastrointestinal tract	• Effect on physiological homeostasis • No overt intestinal phenotypes in mice with intestine-specific deletion of Yap (Villin-Cre; Yap^flox/flox^) (Cai et al., 2010) or dual deletion of Yap and Taz (Azzolin et al., 2014) • Suppressed proliferation of crypt cells in adult mice overexpressing a dominant negative form of Tead2 (Imajo et al., 2015) • Important for intestinal regeneration after some types of tissue injury • Severely impaired intestinal regeneration in Villin-Cre; Yap^flox/flox^ mice subjected to dextran sodium sulfate-mediated colitis model (Cai et al., 2010) • Crypt hyperplasia and overgrowth of the small intestine and colon in Villin-Cre; Yap^flox/flox^ mice after injury by whole-body irradiation (Barry et al., 2013)
Heart	• Effect on physiological homeostasis • Dilated cardiomyopathy and premature death in αMHC-Cre; Yap^flox/flox^ mice (Xin et al., 2013) • These cardiac phenotypes might be caused by the absence of Yap in prenatal and postnatal heart development • Important for regeneration after cardiac damage • Compromised cardiac regeneration following myocardial infarction (MI) in α-MHC-Cre; Yap^flox/+^ mice (Del Re et al., 2013) • Improved cardiac regeneration and contractility after MI in mice overexpressing constitutively active form of Yap (Xin et al., 2013)
Kidney	• May be indispensable for physiological homeostasis • Potential role in maintaining the integrity of the glomerular filtration barrier observed in Podocin-Cre; Yap^flox/flox^ (Schwartzman et al., 2016) • High Yap protein expression in podocyte nuclei of adult mouse kidney under normal physiologic conditions (Campbell et al., 2013)
Lung	• Important for in regulation of lung regeneration and resolution of inflammation post injury or infection (LaCanna et al., 2019)
Skin/pancreas	• Appears dispensable for physiological homeostasis
Reproductive organs and development	• TEAD, YAP, and TAZ expression in human reproductive tissues (https://www.proteinatlas.org) • Impaired pregnancy-induced mammary tissue growth in MMTV-Cre; Yap^flox/flox^ mice (Chen et al., 2014) • Human genetic disorders and abnormal developmental phenotypes (including embryonic lethality) in mice with germline deletion of key Hippo pathway effectors

### Phenotypes of Germline Systemic Knockout Mouse Models

As previously mentioned, embryonic lethality was observed upon germline systemic deletion of Yap, Tead1, or Tead4. Homozygous deletion of Yap in mice resulted in embryonic lethality at E8.5 due to yolk sac vasculogenesis and failure of attachment between the allantois and the chorion (Morin-Kensicki et al., 2006). In contrast, mice with heterozygous Yap deletion were viable, fertile, and exhibited no overt abnormalities (Morin-Kensicki et al., 2006). Homozygous deletion of Taz in mice resulted in multicystic kidneys (Hossain et al., 2007; Makita et al., 2008) and diffuse emphysematous changes in the lung (Makita et al., 2008). Partial lethality started at the perinatal stage, and only 20–65% of Taz knockout mice survived to adulthood with smaller body size (Makita et al., 2008) and progressive renal changes (Hossain et al., 2007; Makita et al., 2008).

Mice with homozygous Tead1 deletion died between E11 and E12 with heart and brain phenotypes (enlarged pericardial cavity, bradycardia, a dilated fourth ventricle in the brain) (Chen et al., 1994). Homozygous deletion of Tead2 in mice exhibited no gross abnormalities and were fertile (Sawada et al., 2008). Mice with dual knockout of Tead1 and Tead2 showed growth retardation and severe morphological changes, including abnormalities in mesoderm patterning, notochord development by E8.5. Neither homozygous deletion of Tead1 nor Tead2 in embryos showed such morphological defects, indicating that Tead1 and Tead2 may have redundant functions in these development processes. No phenotypic evaluation has been reported for Tead3 deletion in mice. Finally, homozygous deletion of Tead4 in mice caused preimplantation defects and did not produce trophoblast stem cells, trophectoderm or blastocoel cavities resulting in a preimplantation lethal phenotype (Yagi et al., 2007).

### Tissue-Specific Conditional Knockout Mouse Models

Due to observed embryonic lethality or developmental phenotypes with germline knockouts, several mouse models with tissue-specific deletion of Hippo pathway effectors, especially Yap, have been generated to elucidate their roles in adult tissues. Overall, mice with tissue- or organ-specific Yap, Taz, dual Yap/Taz, or Tead deletion (or mutant overexpression) have indicated that Hippo signaling may be dispensable for physiological homeostasis in at least some tissues, but is critical for regeneration upon tissue damage (Tables 1, 2). Thus, there appears to be a higher risk in patients with underlying comorbidities and/or insults caused by therapeutic agents and/or comedications. In the case of developing small molecules inhibiting the Hippo pathway, it will be important to minimize off-target activity in order to achieve efficacious exposures, minimize potential tissue injury, and widen the therapeutic window. Here, we provide a brief overview of such mouse models and discuss potential safety concerns and de-risking strategies (Tables 1, 2).

**TABLE 2:** Summary of potential safety concerns and mitigation strategies and challenges.

**Potential safety concern**	**Data**	**Mitigation strategies and challenges**
Potential side effects on normal tissue functions and homeostasis	• Widely expressed • Typically associated with stem and progenitor cell expansion, cell survival, and inhibition of differentiation; inhibition of YAP/TAZ has the opposite effects • Crosstalk with other developmental signaling pathways • Appears dispensable for homeostasis at least in some tissues • No data from animal models with systemic deletion of Yap, Taz, and/or Teads at a chosen time in adulthood	• Conduct phenotyping studies using mice with systemic knockout or knockdown of Yap, Taz, and/or Teads at a chosen time in adulthood • Generate mouse models for systemic deletion of all four Teads in adult mice • Characterize tool compounds and/or early lead molecules in preclinical toxicity studies
Impact on patients’ ability to recover from tissue injury	• Essential for regeneration across tissues, including liver, gastrointestinal tract, and heart	• Minimize off-target toxicities • Challenging to address in preclinical toxicity studies • Set appropriate patient inclusion/exclusion criteria in clinic

### Liver

Liver-specific overexpression of a dominant-negative form of Tead2 in mouse did not affect normal liver homeostasis, and there were no significant differences in liver size, histology, and expression levels of Yap target genes (*Afp, Birc5/survivin, Ctgf, Epcam, Opn, c-Myc, Gpc3*, and *Sox4*) (Liu-Chittenden et al., 2012). Similarly, no notable hepatic changes were observed in adult mice with liver-specific deletion of Yap (polyC injected-Mx1-Cre; Yap^flox/flox^) (Bai et al., 2012). However, when the same mice were subjected to bile duct ligation (BDL), they developed ascites, and 35% of animals died within 15 days, while all control littermates showed no ascites or mortality (Bai et al., 2012). Liver histology revealed compromised bile duct proliferation, hepatocyte necrosis, and delayed hepatocyte proliferation (Bai et al., 2012), indicating that Yap mediates the bile duct and hepatocyte reaction after injury. In agreement with these findings, in human livers obtained from patients with chronic cholestasis, nuclear YAP protein expression was increased in the bile ductular reactions (Bai et al., 2012).

Similar to compromised hepatic responses following injury reported by Bai et al. (2012), mice with liver-specific deletion of both Yap and Taz (Albumin-Cre; Yap^flox/flox^; Taz^flox/flox^) showed less efficient liver regeneration following partial hepatectomy (Lu et al., 2018). Interestingly, these mice showed increased liver size and liver injury (hepatic macrophages in area of necrosis and increased ALT/AST) at 2 months as well as adenoma formation at 12 months of age (Lu et al., 2018). The liver condition observed at 2 months of age appears to be associated with impaired bile duct functions rather than impaired homeostasis. This correlates with previous literature demonstrating a critical role of Yap in biliary development (Yimlamai et al., 2014) and bile duct proliferation following injury (Bai et al., 2012).

### Gastrointestinal Tract

The gene transfer of a dominant-negative form of Tead4 into intestinal epithelium in adult mice resulted in suppressed crypt cell proliferation and decreased crypt base columnar cells (Imajo et al., 2015). In contrast, under normal homeostasis, mice with intestinal-specific Yap deletion (Villin-Cre; Yap^flox/flox^) (Cai et al., 2010) or dual deletion of Yap and Taz (Azzolin et al., 2014) showed no significant differences in crypt cell proliferation or intestinal defects compared to wild-type controls.

When mice with intestine-specific Yap deletion (Villin-Cre; Yap^flox/flox^) were subjected to a dextran sodium sulfate (DSS)-induced colitis/injury model, a dramatic increase in mortality rate and rapid decrease in body weight were noted (Cai et al., 2010). They also demonstrated substantial histopathologic damage with significant loss of crypts, scattered colonic epithelial cells with fewer proliferating cells, and increased apoptotic cells. These results suggest that while Yap is largely dispensable for normal intestinal homeostasis, it is critical for DSS-induced crypt regeneration. In contrast, the same mouse model showed crypt hyperplasia and overgrowth throughout the small intestine and colon following injury by whole-body irradiation (Barry et al., 2013), suggesting that Yap has a growth-suppressive function. These results indicate the function of Yap during intestinal regeneration could depend on the injury type. Wnt signaling is known to be critical during intestinal regeneration following irradiation injury (Davies et al., 2008; Ashton et al., 2010). Indeed, crypts in Villin-Cre; Yap^flox/flox^ mice displayed upregulation of the Wnt target genes, CD44 and SOX9, following irradiation (Barry et al., 2013), indicating that Yap suppresses intestinal stem cell proliferation by inhibiting Wnt signaling.

### Heart

Cardiac phenotypes associated with deletion of various Hippo pathway members, including Yap and Taz have been reviewed elsewhere (Zhou et al., 2015). Several mouse models with heart-specific deletion of Yap resulted in embryonic lethality or perinatal lethality with cardiac phenotypes, indicating that the Hippo pathway is essential for heart development.

Using the α-myosin heavy chain (αMHC) as a promoter of Cre rescued embryonic or perinatal lethality, and no abnormal phenotypes were observed at birth; however, they became lethargic with labored breathing and died between 11 and 20 weeks of age (Xin et al., 2013). Although cardiac size and structure were normal in αMHC-Cre; Yap^flox/flox^ mice at 3 weeks of age, thinning of the ventricular walls and dilated cardiomyopathy were noted by 9 weeks of age, which worsened with age and culminated in atrial thrombosis, fibrosis, and lethal heart failure. These cardiac phenotypes in adult mice might be caused by impaired cardiac development rather than impaired homeostasis. This is because (1) αMHC-Cre; is up-regulated during fetal and postnatal cardiac development (Lyons et al., 1990); (2) Yap1 protein was robustly detected in neonatal and juvenile mouse heart and declined with age, and it was nearly undetectable by 12 weeks of age (von Gise et al., 2012); and (3) Yap1 was localized predominantly in the cytoplasm of cardiomyocytes in adult mice under normal physiological conditions (Del Re et al., 2013). Therefore, cardiac phenotypes observed in adult αMHC-Cre; Yap^flox/flox^ mice might be related to the role of Yap in prenatal and postnatal heart development. In contrast to αMHC-Cre; Yap^flox/flox^ mice, heterozygous deletion of Yap in heart (αMHC-Cre; Yap^flox/+^) did not cause any overt cardiac phenotype, including cardiac size or functions assessed by echocardiography (Del Re et al., 2013).

In wild-type mice, after myocardial injury (MI, caused by permanent ligation of the left descending coronary artery), Yap1 was predominantly localized in the nuclei in the border zone but not in the remote region, indicating that a subpopulation of Yap1 is selectively activated at the site of injury during MI (Del Re et al., 2013). Following MI, αMHC-Cre; Yap^flox/+^ showed decreased cross-sectional area of cardiomyocytes (possible blunted hypertrophic response), increased infarct size (suggestive of impaired healing), increased fibrosis and apoptosis, and reduced proliferation (Del Re et al., 2013). Echocardiography studies indicated impaired myocardial functions, enlarged ventricular chambers, and significantly thinner posterior walls. Conversely, expression of a constitutively active form of Yap (S112A) in the adult heart stimulated cardiac regeneration and improved contractility after MI (Xin et al., 2013). These results indicate that Yap plays a role in stem cells or cardiac progenitor cells contributing to regeneration in response to injury. This is consistent with results that conditional deletion of upstream components (Lats1/2 and Sav1) displayed increased renewal of adult cardiomyocytes after apex resection or MI (Zhou et al., 2015).

Cardiac-specific conditional deletion of Taz (αMHC-Cre; Taz^flox/flox^) were viable and did not display any obvious cardiac defects until they were combined with heterozygous deletion of Yap (Xin et al., 2013), indicating that Taz may be dispensable during perinatal or postnatal heart development. Interestingly, adult human and mouse hearts had more TAZ than YAP1 by mRNA and protein expression and their increased expression in diseased hearts were proportional (Hou et al., 2017).

### Kidney

Developmental phenotypes observed upon conditional deletion of Hippo pathway effectors, Yap and/or Taz, in the progenitor cap mesenchyme population of the developing kidney (Six2-Cre; Yap^flox/flox^, Six2-Cre; Taz^flox/flox^, or Six2-Cre; Yap^flox/flox^; Taz^flox/flox^, respectively) have been reviewed elsewhere (Varelas, 2014). In contrast to Yap/Taz deletion in Six2 progenitor cells, mice with podocyte-specific Yap deletion (Podocin-Cre; Yap^flox/flox^) did not have glomerular or tubulointerstitial abnormalities at birth. However, they developed chronic proteinuria, which correlated with increased podocyte apoptosis and podocyte depletion (Schwartzman et al., 2016). Under normal physiologic conditions, Yap is highly expressed in podocyte nuclei of adult mouse kidney (Campbell et al., 2013). Overall, the results from the various mouse models suggest that Yap is essential for maintaining the integrity of the glomerular filtration barrier in adult kidney by inhibiting podocyte apoptosis (Schwartzman et al., 2016).

### Skin

Although epidermis-specific Yap deletion (K14-Cre; Yap^flox/flox^) resulted in perinatal lethality (Schlegelmilch et al., 2011), adult mice with tamoxifen-induced inactivation of Yap/Taz in epidermal keratinocytes (K14-CreER; Yap^flox/flox^; Taz^flox/+^) appeared phenotypically normal, and their epidermis was histologically indistinguishable from wild-type controls (Zanconato et al., 2015).

### Lung

Mice with germline deletion of murine Taz have abnormal phenotypes associated with lung development (Hossain et al., 2007; Makita et al., 2008). Additionally, recent papers have highlighted a key role of Yap and Taz in regulation of lung regeneration and resolution of inflammation post injury or infection (LaCanna et al., 2019; Sun et al., 2019). These studies are based on deletion of Yap/Taz in relevant cell types in adult mice, thereby bypassing their role in development. Specifically, given that deletion of Taz exacerbates lung fibrosis in mouse models upon injury, this would constitute an additional safety consideration, especially in patient populations with frequent underlying comorbidities or pre-existing conditions.

### Mammary Glands, Pancreas, and Nervous System

Dynamic changes of Yap expression levels and patterns have been reported during mammary gland development in the pregnancy-lactation cycle (Chen et al., 2014). In virgin mouse mammary glands, Yap protein was predominantly localized in nuclei in myoepithelial and cap cells, while the luminal cells displayed uniform intracellular distribution. In mammary glands of pregnant mice, Yap was upregulated in proliferating alveolar cells with prominent nuclear localization. During lactation, Yap was significantly decreased in the alveoli, with only a few scattered cells positive for Yap. Involuted mammary glands showed Yap expression pattern similar to virgin mammary glands. Mice with mammary-specific deletion of Yap (MMTV-Cre; Yap^flox/flox^) had no defects in terminal end bud formation, ductal growth, or ductal branching. However, Yap-deficient mammary glands revealed significantly reduced alveolar structures at 16.5 and 18.5 days of pregnancy, suggesting that Yap promotes mammary epithelial cell survival during pregnancy.

Although robust nuclear localization of YAP protein was detected in normal human and mouse pancreatic tissues, it appeared dispensable for maintaining tissue homeostasis. Pancreata in mice with pancreatic epithelium-specific deletion of Yap (p48-Cre; Yap^flox/flox^) were histologically and functionally indistinguishable from wild-type controls including no apparent differences in their ability to modulate glucose levels (Zhang et al., 2014).

There have been several studies investigating the role of Hippo pathway in brain development or neurogenesis using animal models (Tremblay and Camargo, 2012; Varelas, 2014; Wang et al., 2017). However, to the best of our knowledge, these have focused on embryonic development and there are no reports discussing its role in the homeostasis or regeneration of adult neuronal tissues.

## Future Perspectives

When one considers therapeutically targeting the Hippo pathway, it is clear that many questions will need to be answered. While potential safety concerns exist, the genetic knockout or pathway knockdown models evaluated to date do not necessarily mimic the pharmacology of therapeutic agents. Moreover, much will be learned about pathway biology in the development and assessment of such pathway modulators. Indeed, it is yet to be determined whether it is pathway inhibition, upregulation, or perhaps even both that might be of therapeutic benefit depending on the disease targeted. Additionally, recent studies have highlighted a key link of the Hippo pathway with angiogenesis and a role in endothelial cells (Boopathy and Hong, 2019). Given that targeting tumor angiogenesis is an important therapeutic strategy in targeting cancers, this would be an additional criterion to evaluate safety findings.

Multiple mouse models with tissue-specific deletion have clearly demonstrated that the Hippo pathway is essential for tissue regeneration capability. While targeting the pathway in cancers, although this is a concern for patients with underlying comorbidities and/or insults caused by therapeutic agents and/or comedications, it is not possible to address this in conventional preclinical toxicity studies. Additionally, the use of preclinical tissue injury models would not be recommended unless there is a clear evidence showing the relevance of the insult to human pathogenesis (Morgan et al., 2017). As a consequence, this may need to be addressed clinically, and appropriate patient inclusion/exclusion criteria might be warranted.

To fully characterize on-target toxicities during drug development, it is particularly important to use pharmacologically relevant preclinical species in terms of target homology, expression/distribution, potency, and biological functions (i.e., pharmacodynamics) in preclinical toxicity studies (Dambach et al., 2016; Brennan, 2017). Hippo pathway components, including YAP, TAZ, and TEADs, are evolutionarily conserved across species. Although eight mammalian YAP isoforms and four mammalian TEAD paralogues are known to exist, the exact isoform(s) or paralogue(s) involved in various physiological conditions across adult tissues or tumor environments is yet to be fully elucidated (Finch-Edmondson et al., 2016; Holden and Cunningham, 2018). This could make the selection of appropriate preclinical species for toxicity studies of Hippo pathway inhibitors challenging, and this should be considered at early stages of drug development.

Significant technological advancements have been made in a variety of *in vitro* approaches for the potential detection of clinical organ toxicity. Bioengineered organ models, including micropatterned cultures, three-dimensional cultures, as well as microphysiological systems (MPS; “liver-on-a-chip”) or interconnecting MPSs (“coupled-organs-on-chips”) have now emerged, which aim to recapitulate an organ’s histological, physiological and functional complexity, as well as dynamic multi-organ signaling in human (Proctor et al., 2017; Brown and Khetani, 2018; Weber et al., 2018). These models could further add value beyond conventional technologies that currently support drug discovery and development and enable us to predict and characterize potential on- and off-target toxicities.

The state of the art in terms of the search for inhibitors has been reviewed elsewhere (Santucci et al., 2015; Crawford et al., 2018; Gibault et al., 2018), but it bears mention that the core of the Hippo pathway, whose role it is to regulate YAP and TAZ activity, are known tumor suppressors. In addition, YAP and TAZ are intrinsically disorganized proteins and interact with their transcriptional signaling complex partners, TEADs1-4, via protein-protein interactions, which are notoriously difficult to target. An effective inhibitor targeting the pathway might require a pan-TEAD inhibitor that blocks all four TEADs, and thus at least eight discrete interactions, and that might well be what is required in order to assess the reversibility and manageability of potential side effects observed *in vivo*, to de-risk the findings from genetic studies, and to evaluate whether or not a viable therapeutic window exists. However, it is certain that the therapeutic opportunities across oncology, regenerative medicine, immunotherapy, and beyond are enticing.

## Author Contributions

All authors contributed to the writing and editing of the manuscript. AD and SK-K conceived and planned the topics to be covered.

## Conflict of Interest Statement

All authors are employees and stockholders of Genentech/Roche.
